# Reproducibility of serum cytokines in an elderly population

**DOI:** 10.1186/s12979-020-00201-0

**Published:** 2020-10-13

**Authors:** Jing Guo, Nicole Schupf, Richard P. Mayeux, Yian Gu

**Affiliations:** 1 Taub Institute for Research in Alzheimer’s Disease and the Aging Brain, Columbia University, New York, NY USA; 2 Department of Neurology, Columbia University, New York, NY USA; 3 Gertrude H. Sergievsky Center, Columbia University, New York, NY USA; 4 Department of Epidemiology, Joseph P. Mailman School of Public Health, Columbia University, New York, NY USA

**Keywords:** Reproducibility, Serum, Cytokines, Elderly population

## Abstract

**Background:**

It is important to assess the temporal reproducibility of circulating cytokines for their utility in epidemiological studies. However, existing evidence is limited and inconsistent, especially for the elderly population.

**Methods:**

Sixty-five elderly (mean age = 77.89 ± 6.14 years) subjects were randomly selected from an existing prospective cohort study. Levels of 41 cytokines in 195 serum samples, collected at three separate visits that were up to 15.26 years apart, were measured by the Luminex technology. The temporal reproducibility of cytokines was estimated by the intraclass correlation coefficient (ICC) calculated using a mixed-effects model. In addition, data analyses were stratified by the median (4.49 years) of time intervals across sample collection. Sensitivity analyses were performed when excluding subjects with undetectable samples.

**Results:**

A total of 23 cytokines were detectable in more than 60% of samples. Fair to good (ICC = 0.40 to 0.75) and excellent (ICC > 0.75) reproducibility was found in 10 (Eotaxin, VEGF, FGF-2, G-CSF, MDC, GM-CSF, TGFα, IP-10, MIP-1β, IL-1RA) and 5 (GRO, IFNγ, IL-17, PDGF-AA, IL-4) cytokines, respectively. The results were not changed dramatically in the stratification and sensitivity analyses.

**Conclusions:**

Serum levels of the selected 15 cytokines measured with Luminex technology displayed fair to excellent within-person temporal reproducibility among elderly population.

## Background

Cytokines are secreted polypeptides or glycoproteins that regulate proliferation, apoptosis, and angiogenesis, and involved in the development and progression of various diseases [1]. Cytokine-mediated intercellular communication is regarded as the main mechanism of crosstalk between immune cells [2]. Cytokine and other inflammatory biomarkers play important roles in the pathophysiology of chronic inflammatory diseases [3], cardiovascular diseases [4], neuroinflammation [5], asthma [6], and cancer [7].

Circulating inflammatory molecules are widely used in epidemiological studies to investigate their association with chronic diseases [8–10]. In some population-based studies, especially the large-scale cohort studies with a long follow-up period, a single measure of blood sample collected at baseline is usually employed to represent the long-term state of inflammation of participants. However, high intra-individual variability of cytokines may lead to a misclassification of inflammatory status and biased association analyses. Therefore, it is important to establish the temporal reproducibility of cytokines when investigating the cytokine-disease associations [11].

Several studies have explored the intra-individual temporal reproducibility of cytokines in humans (see Additional file 1: Table S1). However, there were some limitations in the previous studies, including small sample size [12–16], assessment of short-term variability [14, 17–20], analysis of a limited number of cytokines [15, 16, 18, 21], or limited to specific populations such as women [11] or with particular physiological status of pregnancy [19]. Furthermore, the temporal reproducibility of cytokines has been predominantly examined for young and middle-aged adults, and the average age of participants was less than 65 years in many previous studies (see Additional file 1: Table S1). As cytokine levels tend to increase with advanced age [18], a phenomenon called inflammageing [22, 23], it is important to evaluate the temporal reproducibility of cytokines in elderly populations.

In the present study, we aimed to investigate the detectability and temporal reproducibility of a large panel of inflammatory biomarkers among 65 elderly (≥ 65 years) subjects, with 3 serum samples each. Results from this study will be useful to select appropriate cytokines as inflammatory biomarkers for elderly population in the future epidemiological studies.

## Methods

### Study design and participants

The Washington Heights-Inwood Community Aging Project (WHICAP) is an ongoing, prospective, population-based cohort study which is conducted to identify risk factors and biomarkers for aging and dementia. The sampling strategies, recruitment and examination methodology of WHICAP have been described previously [24, 25]. Participants in WHICAP were socioeconomically and racially diverse, community residents in northern Manhattan, aged ≥65 years, and fluent in English or Spanish. At baseline and at the follow-up visits every 18–24 months, participants received comprehensive medical, physical, neurological and neuropsychological examinations. Blood samples were collected at the enrollment and the follow-up visits.

For the current study, we selected a total of 65 participants from the second and third waves of WHICAP who met the following criteria: (a) ≥ 3 blood samples donated at different visits, (b) large number of aliquots left, (c) without history of diagnosed dementia, and (d) complete information on ages at blood draw, sex, race/ethnicity and years of education. To ensure individuals with certain demographic characteristics to be included in this sample, we randomly selected subjects from each of the 24 strata, defined by sex (male vs. female), race/ethnicity (White, Black, Hispanic), waves (1999 vs. 2009), and age groups (< 80 vs. ≥80 years). For each selected individual, we selected one serum sample from the baseline, the most recent, and a middle visit. With three repeated samples each for a total of 65 subjects, we had 80% power to detect acceptable ICC of ≥0.55 with narrow 95% confidence interval (CI) (< ±0.13), or to detect acceptable ICC of ≥0.40 with narrow 95% CI (< ±0.15).

### Cytokine quantification

Peripheral venous blood samples were collected from the participants at the time of their health examination, and were stored at − 80 °C. Never-thawed serum samples were packed in dry-ice and sent to the laboratory for analysis. A panel of 41 inflammatory biomarkers were selected according to the results of previous literatures (see Additional file 1: Table S1), biological functions of cytokines, and availability of commercial assay kits, and were analyzed by the Luminex technology. The magnetic bead-based sandwich immunoassays for cytokines using the MILLIPLEX Human Cytokine Panel 1 (HCYTOMAG-60 k) (MilliporeSigma, St. Louis, MO) were performed according to the manufacturer’s instructions. The serum samples (25 μL) were analyzed in duplicate wells using a Luminex 200 (Luminex Corp, Austin, TX). The cytokine concentrations were determined by Luminex xPONENT v4.2 and MILLIPLEX Analyst v5.1 using 5-p log analysis. All assays were performed using the same lot of Luminex reagents. All samples from the same subject are always in the sample plate. Two sets of quality controls spiked in serum, provided by the manufacturer, were run in duplicate across the plates. This is an ideal substitute for the pool samples since the sample type in this study was also serum. The inter-assay precision is excellent with a mean coefficient of variation (CV) of 6.74% (range: 1.87 to 16.03%), well below the 20% accepted cut off for a ligand-binding assay suggested by the Bioanalytical Method Validation Guidelines for Industry in 2018 [26].

Samples with cytokine levels less than the lower limit of detection (LLOD) were assigned a value of LLOD divided by the square root of two [19]. Observations beyond the upper limit of detection (ULOD) were substituted with ULOD for data analysis. Samples with values between the LLOD and ULOD were regarded as detectable for specific cytokines. The cytokines were retained for further analyses when the proportions of detectable values were at least 60% among all the 195 blood samples [11].

### Statistical analysis

Due to the skewed distribution, cytokine levels were natural logarithm (log)-transformed for approximately normal distribution. The characteristics of subjects were presented as mean [standard deviance (SD)] and number [proportion (%)] for continuous and categorical variables, respectively. The repeated measures correlation (95% CI) between different cytokines were estimated with the R package “rmcorr” which accounts for non-independence among measurements from the same subject [27].

The intraclass correlation coefficient (ICC) was employed to quantify the temporal reproducibility of cytokines. The mixed-effects model, fitted by the R package “lme4” (version 1.1.21) [28], was established when using the levels of each cytokine as dependent variable, setting the random intercept on the repeated measures, and adjusting for the covariates of age at recruitment (years), sex (male, female), race/ethnicity (non-Hispanic White, non-Hispanic Black, Hispanic) and time intervals across blood collection (years). Based on the results of mixed-effects model, ICC was calculated by dividing the between subject variance by the total variance [29]. The model-based parametric bootstrap for mixed models was used to estimate the 95% CI of ICC. For the main analyses, ICC was calculated using all three repeated measurements. The ICC levels of < 0.40, 0.40 ~ 0.75 and > 0.75 mean poor, fair to good and excellent reproducibility, respectively [30].

To examine the impact of time intervals on the cytokine reproducibility, data analyses were performed by the stratification of median of time intervals between the 1st and 3rd measures. Besides, we also repeated analyses by excluding samples with undetected values (< LLOD or > ULOD), or by additionally adjusting for the baseline comorbidities including hypertension, diabetes and cardiovascular diseases, or by additionally adjusting for the body mass index (BMI, kg/m^2^) categories (normal weight [< 25], overweight [≥ 25 and <  30], and obese [> 30]) at each visit, or by additionally adjusting for the time of day (ante meridiem or post meridiem) of blood sample collection. Sensitivity analyses were also conducted by the stratification of sex.

All the data analyses were performed with R (version 3.6.1).

## Results

### Characteristics of subjects

Participant characteristics are presented in Table 1. Among the 65 subjects, the mean of age at the collection of first blood sample was 77.89 (SD = 6.14; range = 67.34 to 90.49) years. By design, about half of the subjects were males, about two thirds were < 80 years and the rest ≥80 years, the White, Black and Hispanic population each accounted for nearly one third of the sample, and the proportions of individuals from the second and third waves of WHICAP were 55.38 and 44.62%, respectively. The average (SD) years of education was 12.26 (4.95). At the collection of first blood sample, subjects at BMI categories of normal weight, overweight, and obese accounted for 26.42, 35.85, and 37.74%, respectively. More than half (58.00%) of the first blood samples were collected in the morning. Participants with hypertension, diabetes, and cardiovascular diseases at baseline accounted for 92.31, 29.23, and 56.92%, respectively. The medians of time intervals were 2.45 [interquartile range (IQR) = 2.00 to 5.72], 1.78 (IQR = 1.63 to 2.19), and 4.49 (IQR = 3.91 to 8.09) years between the 1st and 2nd, between the 2nd and 3rd, and between the 1st and 3rd blood samples, respectively.

**Table 1:** Characteristics of participants across three repeated measurements

Characteristics	The 1st measurement	The 2nd measurement	The 3rd measurement
Sample size	65	65	65
Age (years), mean (SD)	77.89 (6.14)	82.50 (4.85)	84.43 (4.85)
65–75, n (%)	23 (35.38%)	5 (7.69)	2 (3.08)
75–80, n (%)	19 (29.23%)	13 (20.00)	9 (13.85)
≥ 80, n (%)	23 (35.38%)	47 (72.31)	54 (83.08)
Gender, n (%)			
Male	32 (49.23)	32 (49.23)	32 (49.23)
Female	33 (50.77)	33 (50.77)	33 (50.77)
Race/ethnicity, n (%)			
White	22 (33.85)	22 (33.85)	22 (33.85)
Black	21 (32.31)	21 (32.31)	21 (32.31)
Hispanic	22 (33.85)	22 (33.85)	22 (33.85)
Education duration (years), mean (SD)	12.26 (4.95)	12.26 (4.95)	12.26 (4.95)
BMI categories, n (%) ^a^			
Normal	14 (26.42)	14 (25.45)	16 (29.09)
Overweight	19 (35.85)	20 (36.36)	22 (40.00)
Obese	20 (37.74)	21 (38.18)	17 (30.91)
Time of day of blood collection, n (%) ^a^			
Ante meridiem	29 (58.00)	37 (57.81)	43 (66.15)
Post meridiem	21 (42.00)	27 (42.19)	22 (33.85)
WHICAP wave, n (%)			
The second wave (1999 cohort)	36 (55.38)	36 (55.38)	36 (55.38)
The third wave (2009 cohort)	29 (44.62)	29 (44.62)	29 (44.62)
Baseline hypertension, n (%)			
No	5 (7.69)	–	–
Yes	60 (92.31)	–	–
Baseline diabetes, n (%)			
No	46 (70.77)	–	–
Yes	19 (29.23)	–	–
Baseline cardiovascular diseases, n (%)			
No	28 (43.08)	–	–
Yes	37 (56.92)	–	–

*SD* standard deviance, *n* number of subjects, *%* proportion, *BMI* body mass index, *WHICAP* Washington Heights-Inwood Community Aging Project.

^a^Sum of frequency of some characteristics was less than 65 at specific measurement due to missing data

### Levels of cytokines

The intra- and inter-assay CV, proportion of detectable samples, and median and range of cytokines were estimated among the 195 specimens from 65 participants (Table 2). The detectable percentage ranged from 4.62 to 100% for the 41 cytokines, of which 23 analytes with detectable percentage ≥ 60% were retained for further assessment on the temporal reproducibility. The results of correlations indicated that some cytokines were significantly correlated with each other (*p* < 0.05) (see Additional file 1: Fig. S1).

**Table 2:** Levels of serum cytokines

Cytokines	Intra-assay CV (%)	Inter-assay CV (%)	Detectable proportions (%) ^a^	Median (P_25_-P_75_) (pg/mL)	Range (pg/mL)
EGF	0.91	7.19	99.49	226.71 (126.59, 374.77)	< 3.17 to 1657
Eotaxin	1.37	3.48	100	134.79 (98.66, 183)	8.46 to 562.15
FGF-2	1.68	7.56	88.72	48.23 (23.39, 105.09)	< 13.05 to 429.02
Flt-3 L	3.17	7.99	**48.21**	3.29 (3.29, 30.1)	< 4.65 to 1769
Fractalkine	2.05	12.69	**43.59**	21.48 (18.72, 61.34)	< 26.48 to > 9834
G-CSF	3.06	8.69	91.28	20.45 (11.17, 49.02)	< 3.97 to 404.65
GM-CSF	7.45	6.89	80.51	7.58 (3.54, 17.48)	< 3.16 to 1287
GRO	1.07	4.65	77.44	2216 (1264, 7288.5)	470.26 to > 9906
IFNα2	5.34	5.92	**55.90**	7.9 (3.23, 25.39)	< 4.57 to 924.48
IFNγ	0.44	8.50	75.38	5.06 (2.07, 11.76)	< 2.04 to 1183
IL-10	5.26	4.49	**25.13**	2.21 (2.21, 3.21)	< 3.13 to 1304
IL-12p40	3.47	6.37	**30.26**	4.79 (4.79, 11.68)	< 6.78 to 605.62
IL-12p70	0.99	2.68	**27.69**	2.02 (2.02, 3.74)	< 2.85 to 655.57
IL-13	3.05	5.86	**26.15**	3.33 (3.33, 6.25)	< 4.71 to 598.37
IL-15	4.94	6.30	**34.36**	2.04 (2.04, 4.3)	< 2.88 to 153.79
IL-17	2.90	4.36	70.77	2.57 (0.73, 7.72)	< 1.03 to 451.19
IL-1α	9.92	5.47	**43.59**	3.6 (3.6, 25.14)	< 5.09 to 3539
IL-1β	5.15	8.15	**53.33**	1.48 (0.88, 4.86)	< 1.24 to 2581
IL-1RA	3.02	8.20	90.26	15.7 (7.66, 31.01)	< 3.65 to 3857
IL-2	3.14	4.48	**28.72**	1.98 (1.98, 3.62)	< 2.80 to 86.71
IL-3	11.71	12.30	**4.62**	1.97 (1.97, 1.97)	< 2.78 to 13.07
IL-4	8.57	7.22	83.51	31.18 (9.96, 79.75)	< 5.42 to 375.29
IL-5	4.03	1.87	**22.05**	0.76 (0.76, 0.94)	< 1.07 to 72.28
IL-6	4.99	6.19	**42.56**	1.32 (1.32, 19.72)	< 1.86 to 7220
IL-7	5.37	4.70	**43.59**	2.64 (2.34, 7.73)	< 3.31 to 119.38
IL-8	2.91	4.22	97.44	36.4 (15.12, 245.41)	3.84 to > 9613
IL-9	3.91	5.98	**15.38**	1.99 (1.99, 1.99)	< 2.81 to 128.69
IP-10	2.69	3.44	100	222.35 (175.6, 307.84)	49.15 to 1009
MCP-1	3.53	5.93	100	590.84 (429.88, 902.12)	118.05 to 9309
MCP-3	0.72	5.50	61.54	6.17 (2.47, 34.91)	< 3.50 to 2891
MDC	9.14	8.11	100	1043 (758.06, 1409)	234.05 to 6725
MIP-1α	8.53	5.06	88.21	19.24 (6.44, 69.77)	< 3.12 to > 5472
MIP-1β	0.65	6.53	99.49	48.04 (28, 89.12)	< 4.50 to 1251
PDGF-AA	2.00	3.91	99.49	3576 (2533.5, 4803.5)	305.27 to > 8786
PDGF-AB/BB	6.91	9.83	**30.77**	9490 (8841, 9490)	3148 to > 9490
RANTES	8.08	12.69	96.92	2730 (1845.5, 3724.5)	619.28 to > 13,438
sCD40L	1.74	6.59	84.10	9529 (4893.5, 13,789)	< 2.86 to > 17,067
TGFα	1.09	16.03	66.15	4.72 (2.18, 8.31)	< 3.09 to > 5120
TNFα	2.78	7.59	95.90	16.48 (9.66, 27.52)	< 2.93 to 716.76
TNFβ	4.15	5.78	**19.49**	2.01 (2.01, 2.01)	< 2.84 to 639.95
VEGF	4.22	7.00	88.21	161.45 (71.86, 326.21)	< 20.50 to 919.48

*CV* coefficient of variation, *P*_*25*_*-P*_*75*_ the 25th to the 75th percentiles, *EGF* epidermal growth factor, *FGF-2* fibroblast growth factor 2, *Flt-3 L* fms-like tyrosine kinase 3 ligand, *G-CSF* granulocyte colony-stimulating factor, *GM-CSF* granulocyte macrophage colony-stimulating factor, *GRO* growth regulated oncogene, *IFNα2* interferon alpha 2, *IFNγ* interferon gamma, *IL* interleukin, *IL-1RA* interleukin-1 receptor antagonist, *IP-10* interferon gamma-induced protein 10, *MCP* monocyte chemotactic protein, *MDC* macrophage-derived chemokine, *MIP* macrophage inflammatory proteins, *PDGF* platelet-derived growth factor, *RANTES* regulated on activation, normal T cell expressed and secreted, *TGF* transforming growth factor, *TNF* tumor necrosis factor, *VEGF* vascular endothelial growth factor, *sCD40L* soluble cluster of differentiation 14 ligand.

^a^Values in bold mean that the proportions of subjects with detectable values (between LLOD and ULOD) were <  60%. A total of 18 cytokines were excluded in the further data analyses due to the low detectable rate

### Temporal reproducibility of cytokines

**Fig. 1:**
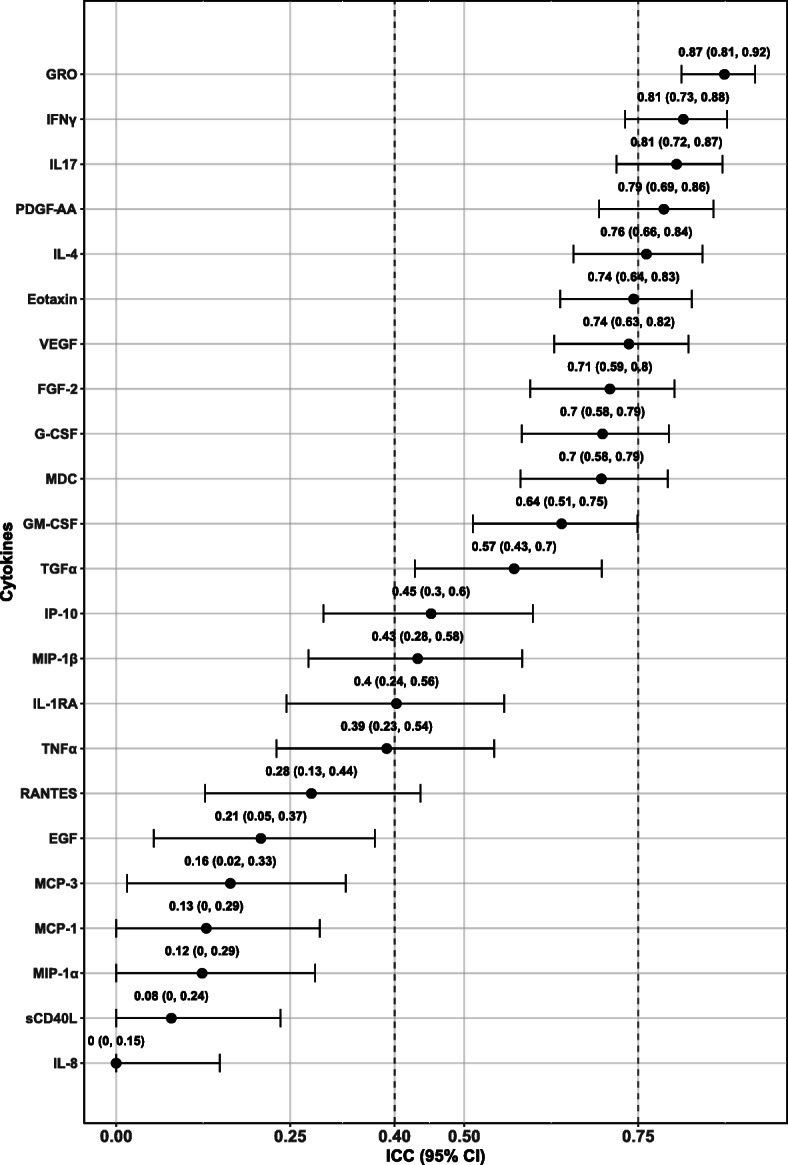
ICC (95% CI) of cytokines. The dots and horizontal bars represent the ICC and corresponding confidence intervals, respectively. ICC was estimated by the mixed-effects model adjusting for age at recruitment, gender, race/ethnicity and time spans across blood collection. The ICC levels of 0.40 ~ 0.75 and > 0.75 are corresponding to fair to good and strong reliabilities, respectively. ICC: intraclass correlation coefficient; CI: confidence interval

The ICC (95% CI) of cytokines measured in all samples collected at three separate visits was ranked and presented in Fig. 1. The lowest ICC (95% CI) was approximated to zero for IL8, and the highest was 0.87 (0.81, 0.92) for GRO. Results showed that the temporal reproducibility was fair to good for 10 cytokines (ICC = 0.40 ~ 0.75; including Eotaxin, VEGF, FGF-2, G-CSF, MDC, GM-CSF, TGFα, IP-10, MIP-1β, IL-1RA), and were excellent for 5 cytokines (ICC > 0.75; including GRO, IFNγ, IL-17, PDGF-AA, IL-4). The remaining 8 cytokines displayed poor temporal reproducibility.

**Fig. 2:**
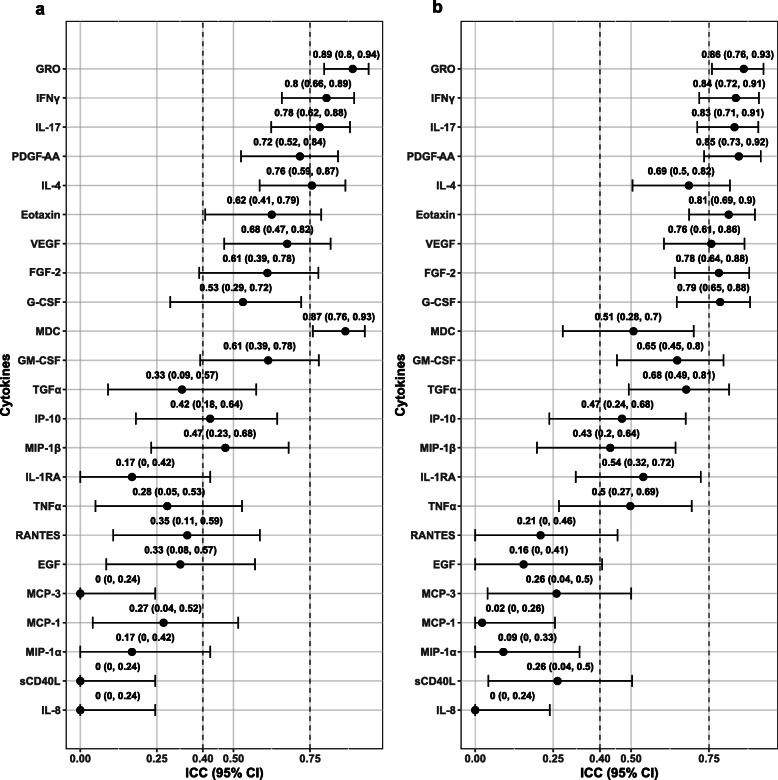
ICC (95% CI) of cytokine levels stratified by the median (4.49 years) of time interval between the 1st and 3rd measures. The dots and horizontal bars represent the ICC and corresponding confidence intervals, respectively. Results of the subjects with time spans < 4.49 years (*n* = 32) and ≥ 4.49 years (*n* = 33) were plotted in the panel (**a**) and (**b**), respectively. ICC: intraclass correlation coefficient; CI: confidence interval

Stratification analyses were additionally conducted, dividing by the median (4.49 years) of time intervals between the 1st and 3rd blood samples (Fig. 2). Compared with the results of the main analyses using all the subjects, the ICCs remained similar except for TNFα, for which the ICC increased into fair to good levels among those with time intervals ≥4.49 years.

After excluding the subjects with undetectable levels of cytokines, for the cytokines with detectable rates less than 100% (Table 2), the results remained similar although in general the ICC reduced slightly except for MCP-3, which the ICC increased (0.41, 95% CI = 0.20 to 0.62) (see Additional file 1: Fig. S2). Robust results were obtained when additionally adjusting for the baseline comorbidities (see Additional file 1: Fig. S3), or for BMI categories (see Additional file 1: Fig. S4), or for the time of day of blood sample collection (see Additional file 1: Fig. S5), except for IP-10 and IL-1RA of which the ICC decreased when adjusting for BMI, and except for TNFα of which ICC increased when adjusting for the time of day of blood sample collection. The ICC levels stratified by sex were similar compared with the main results of all subjects, except that the ICC of TNFα increased into fair to good levels in males (see Additional file 1: Fig. S6).

## Discussion

In the present study of 65 elderly subjects from WHICAP study, we assessed the temporal reproducibility of serum cytokines in samples collected at three separate visits over a relatively long period of time. The results revealed that 15 out of 41 cytokines measured by the Luminex technology were detectable in over 60% of serum samples and had good reproducibility (ICC > 0.40), indicating that a single measurement of these biomarkers well captures the within-individual average level over a long period of time.

By definition, ICC measures the inter-person variability relative to the total variability [29]. The high values of ICC indicated that the variation of cytokine levels was considerably higher between subjects than within subjects, which supports the good reproducibility of these biomarkers over a long term. Therefore, cytokines having high ICC can be used in the epidemiological studies focusing on the associations between one-time measured cytokines and diseases.

The temporal reproducibility of circulating cytokines measured by multiplex immunoassays or ELISA has been reported previously. Our findings are consistent with previous studies, supporting good to excellent ICC for IL-17 [17], Eotaxin [11], G-CSF [12, 14, 17], MIP-1β [14, 17] and IL-1RA [11]. However, inconsistencies also exist, which might be due to the discrepancies in study design, sample size, type of specimen, and assay methods [18, 31]. For example, extremely low ICC of IL-8 was found in our study (near to 0) and other studies (from 0.02 to 0.05) [11, 14] in which cytokine levels were examined in serum. Conversely, a good reproducibility of IL-8 has been demonstrated in plasma (> 0.40) [12, 14, 18]. It has been reported that the concentrations of cytokines and the corresponding ICC are higher in plasma than in serum [14, 31, 32]. Levels of some cytokines can be different between matched specimens of plasma and serum collected from the same individual, and also can be affected by different anticoagulants used in plasma samples, suggesting the important roles of specimens and anticoagulants in the measurement of cytokines [33]. As a result, different ICC values of a same cytokine reported by different studies may also be caused by various specimens and anticoagulants used.

The circulating levels of cytokines are reported to be influenced by various factors including demographics (age, gender, race/ethnicity), life styles (exercise, sleep, smoking, drinking, diet and adiposity), medical treatment and circadian physiological variability [11, 18, 34–36]. Therefore, the inconsistency might also be partially due to the discrepancies in characteristics of the study population, and time periods across measurements [18, 31]. For instance, compared with the reproducibility of IL-4 (ICC = 0.76) in our study, similar results were observed among the healthy women from Sweden (ICC = 0.70) [12] and USA (ICC = 0.92) [11], but lower ICC values (< 0.40) were reported in populations from other areas [17, 18]. We found an excellent reproducibility of IFNγ (ICC = 0.82) in the elderly subjects. In contract, lower values of ICC for IFNγ (about 0.50) have been obtained from the younger populations for which the mean age was less than 60 years [17, 18, 37]. Data from laboratory studies indicate that the secretion of IFNγ, a pro-inflammatory cytokine mainly produced by the type 1 T cells, and the IFNγ inducible inflammation cascade are increased with aging [38, 39]. Thus, inconsistent reproducibility of IFNγ between current and previous studies was supported by the aging effects.

An assessment of the long-term reproducibility of biomarkers is essential to explore the associations between biomarkers and diseases with long etiological windows, such as the neurodegenerative disease, cardiometabolic disease, or cancer. Majority of the previous studies evaluating the reproducibility of cytokines had used samples spanning a shorter period of time, such as 14 days [17], several months [14, 15, 18, 19, 21], 1 ~ 2 years [16], or 2 ~ 3 years [11, 12, 37]. The current study has a median time span of 4.49 (range = 2.86 to 15.26) years between the first and the last samples, probably fitting better the goal of evaluating the long-term reproducibility of cytokines. Only two previous studies covered similar (over 5 years) [13] or markedly longer (median = 18.3 years) [30] periods of time. However, the generalization of results in the two previous studies might be precluded due to the small sample size (*n* = 28) [13] or restriction to males [30].

With increasing time intervals, the temporal reproducibility of G-CSF kept good to excellent over 14 days (ICC = 0.50) [17], 7 months (ICC = 0.73) [14], 1 ~ 3 years (ICC = 0.75) [12] and ≥ 4.49 years (ICC = 0.79, the present study). Meanwhile, previous studies showed inconsistent trends of temporal reproducibility of some cytokines within a fixed population [15, 30]. The ICC of TNFα was reported to be diminished from 0.54 to 0.49 with increased time intervals from 0 ~ 1.9 to ≥15 years, respectively, in males whose median age was 45.6 years [30]; however, it was reported to be increased from 0.39 to 0.47 for time spans of 6 weeks and 9 months, respectively, in the combination of males and females whose mean age was 64 years [15]. In line with the ICC trends of the second study mentioned above [15], we found a lower temporal reproducibility of TNFα in subjects with time spans < 4.49 years (ICC = 0.28) compared with those with longer time intervals (≥ 4.49 years, ICC = 0.50). The ICC trends over different durations may be influenced by various factors including the physiological homeostasis, storage conditions, degradation rate, pre-analytical processing, and other characteristics which can influence the biomarker levels and are changeable within a subject [30, 40]. Further studies are needed to examine the effect of duration between samples on cytokine reproducibility.

In the sensitivity analyses without undetectable values, we found similar, although slightly reduced, temporal reproducibility for most cytokines except for MCP-3 of which the ICC increased (see Additional file 1: Fig. S2). The reduction of ICC might be due to the exclusion of subjects who had consistently low or undetectable levels of analytes across different samples. On one hand, the undetectable values of cytokines mean imprecise measurements. On the other hand, the consistently undetectable levels can reflect acceptable temporal reproducibility within a subject, implying potential usefulness of these undetectable levels. For instance, subjects with consistently low or undetectable values of cytokines can be categorized into the low “exposure” group in the epidemiological studies. The impact of undetectable biomarkers was limited in our study because cytokines with inadequate detection rates (< 60%) were excluded in the primary analyses [11, 14]; and similar results were observed when additionally removing the subjects with undetectable values of cytokines (see Additional file 1: Fig. S2).

Results from previous studies demonstrated sex differences in levels of some cytokines among young adults (median age = 22 years) [41], and in cytokine secretion responsiveness of lymphomonocytes under stress conditions [42]. We found nonsignificant differences of cytokine levels (data not shown, *p* > 0.10) and similar reproducibility of cytokines between older males and females in this study, which might be due to the depletion of sex steroid hormones in elderly subjects. Although it has been reported that BMI is significantly correlated with levels of cytokines in cerebrospinal fluid [43], and that circadian clock is a regulator of cytokines [36], we found robust ICC of serum cytokines when additionally adjusting for BMI or time of day of blood sample collection, indicating limited influence of BMI and time of day of blood drawn on the reproducibility of cytokines in the present study.

There were several advantages in this study. Few studies have examined the elderly population previously. Our study was performed among subjects with a mean age of 77.89 years, which provided useful information for the studies focusing on the health effects of cytokines among elderly individuals. In addition, a large panel of cytokines (41 in total) were analyzed in the present study, while fewer (no more than 20) cytokines were investigated in some previous studies [13, 15–19, 21, 37]. Additionally, because subjects in this study were racially diverse and community-sourced, our findings can be widely generalized.

Some limitations should be noted. Although this study represent one of the largest study in terms of number of cytokines measured, a total of 41 cytokines may still not give a comprehensive insight into the overall inflammatory profile, which may involve more cytokines [44, 45]. Besides, cytokine ICC could not be estimated by stratification of race due to the limited sample size. Secondly, while subjects in this study were limited to those without dementia, the influences of other diseases could not be fully excluded. However, the results were similar when adjusting for the history of hypertension, diabetes and cardiovascular diseases, suggesting limited influence of these comorbidities. Although incident diseases developed in the midst of blood collection intervals may disrupt the cytokine homeostasis and enhance the variability of cytokine levels, inducing the underestimation of cytokine ICC, it is still reasonable to use the selected cytokines with good reproducibility in the future epidemiological studies to investigate the cytokine-associated diseases. Thirdly, the disparity in time intervals might have induced biased estimation of ICC. To overcome this issue, the ICC was calculated with adjustments of time intervals. We also found robust results after a series analyses within subgroups with shorter intervals between samples, suggesting limited impacts of time intervals on the temporal reproducibility of selected cytokines. Fourthly, the results should be interpreted with caution for the cytokines of which the point estimation of ICC was more than the cut-off value of 0.40 but the confidence intervals included 0.40 (IP-10, MIP-1β, IL-1RA).

## Conclusions

In conclusion, the present study demonstrated a good temporal reproducibility of 15 serum cytokines (Eotaxin, VEGF, FGF-2, G-CSF, MDC, GM-CSF, TGFα, IP-10, MIP-1β, IL-1RA, GOP, IFNγ, IL-17, PDGF-AA, IL-4) which were measured by the multiplex technology. This suggests that a single measurement of the selected cytokines is likely to be suitable for characterizing the immune and inflammation status over a long period in the prospective epidemiological studies, especially for the elderly population.

## Supplementary information

**Additional file 1:** **Table S1.** Assessments on the temporal reproducibility of circulating cytokines in previous studies. **Fig. S1.** Paired repeated measures correlation among different cytokines. **Fig. S2.** ICC (95% CI) of cytokines by excluding the subjects with undetectable values. **Fig. S3.** ICC (95% CI) of cytokines when additionally adjusting for baseline comorbidities. **Fig. S4.** ICC (95% CI) of cytokines when additionally adjusting for BMI categories. **Fig. S5.** ICC (95% CI) of cytokines when additionally adjusting for time of day of blood sample collection. **Fig. S6.** ICC (95% CI) of cytokines stratified by sex.

## Data Availability

The data that support the findings of this study will be made available from the authors upon reasonable request and with permission of the WHICAP Study Committee.

## Abbreviations

BMI: Body mass index
CI: Confidence interval
CV: Intra-assay coefficient of variation
EGF: Epidermal growth factor
FGF-2: Fibroblast growth factor 2
Flt-3 L: Fms-like tyrosine kinase 3 ligand
G-CSF: Granulocyte colony-stimulating factor
GM-CSF: Granulocyte macrophage colony-stimulating factor
GRO: Growth regulated oncogene
ICC: Intraclass correlation coefficient
IFNα2: Interferon alpha 2
IFNγ: Interferon gamma
IL: Interleukin
IL-1RA: Interleukin-1 receptor antagonist
IP-10: Interferon gamma-induced protein 10
IQR: Interquartile range
LLOD: Lower limit of detection
MCP: Monocyte chemotactic protein
MDC: Macrophage-derived chemokine
MIP: Macrophage inflammatory proteins
PDGF: Platelet-derived growth factor
RANTES: Regulated on activation, normal T cell expressed and secreted
SD: Standard deviance
TGF: Transforming growth factor
TNF: Tumor necrosis factor
ULOD: Upper limit of detection
VEGF: Vascular endothelial growth factor
WHICAP: Washington Heights-Inwood Community Aging Project
log: Natural logarithm
sCD40L: Soluble cluster of differentiation 14 ligand
