# Supplementary material for: Reproducibility of serum cytokines in an elderly population
Source: Immun Ageing. 2020 Oct 13;17:29. doi: 10.1186/s12979-020-00201-0 (PMC7556943; doi:10.1186/s12979-020-00201-0)
Supplement: Supplementary file 1 — Additional file 1: Table S1. Assessments on the temporal reproducibility of circulating cytokines in previous studies. Fig. S1. Paired repeated measures correlation among different cytokines. Fig. S2. ICC (95% CI) of cytokines by excluding the subjects with undetectable values. Fig. S3. ICC (95% CI) of cytokines when additionally adjusting for baseline comorbidities. Fig. S4. ICC (95% CI) of cytokines when additionally adjusting for BMI categories. Fig. S5. ICC (95% CI) of cytokines when additionally adjusting for time of day of blood sample collection. Fig. S6. ICC (95% CI) of cytokines stratified by sex. [file 12979_2020_201_MOESM1_ESM.docx]

# **Supplementary materials**

Jing Guo^1^, Nicole Schupf^1,2,3,4^, Richard Mayeux^1,2,3,4^, Yian Gu^1,2,3,4*^

^1^ Taub Institute for Research in Alzheimer’s Disease and the Aging Brain, Columbia University, New York, NY, USA

^2^ Department of Neurology, Columbia University, New York, NY, USA

^3^ Gertrude H. Sergievsky Center, Columbia University, New York, NY, USA

^4^ Department of Epidemiology, Joseph P. Mailman School of Public Health, Columbia University, New York, NY, USA

* Corresponding author.

Yian Gu, Ph.D.

[yg2121@cumc.columbia.edu](mailto:yg2121@cumc.columbia.edu)

630 West 168th Street, P&S Box 16

New York, NY 10032

Phone: 212-305-6684

Fax: 212-342-1838

| Contents of supplementary materials | |
| --- | --- |
| Page | Titles of tables and figures |
| 2-7 | **Table S1.** Assessments on the temporal reproducibility of circulating cytokines in previous studies. |
| 8 | **Figure. S1.** Repeated measures correlation among different cytokines. |
| 9 | **Figure. S2.** ICC (95% CI) of cytokines by excluding the subjects with undetectable values. |
| 10 | **Figure. S3.** ICC (95% CI) of cytokines when additionally adjusting for baseline comorbidities. |
| 11 | **Figure. S4.** ICC (95% CI) of cytokines when additionally adjusting for BMI categories. |
| 12 | **Figure. S5.** ICC (95% CI) of cytokines when additionally adjusting for time of day of blood sample collection. |
| 13 | **Figure. S6.** ICC (95% CI) of cytokines stratified by sex. |

| Table S1. Assessments on the temporal reproducibility of circulating cytokines in previous studies. | | | | | | | | | | |
| --- | --- | --- | --- | --- | --- | --- | --- | --- | --- | --- |
| Study | **Country/region & study name** | **Sample size of participants** | **Age at baseline (years)** | **Participants** | **Specimens** | **Storage** | **Cytokines** | **Test methods** | **Number of repeated measurements** | **Time intervals** |
| This study | USA;  The Washington Heights Inwood Community Aging Project (WHICAP) | 65 | Range=67.34 to 90.49; Mean=77.89 | - Elderly subjects without dementia; - Males: n=32;   Female: n=33 | Serum | -80 °C | - N=41; - EGF, Eotaxin, FGF-2, Flt-3L, Fractalkine, G-CSF, GM-CSF, GRO, IFNα2, IFNγ, IL-10, IL-12p40, IL-12p70, IL-13, IL-15, IL-17, IL-1α, IL-1β, IL-1RA, IL-2, IL-3, IL-4, IL-5, IL-6, IL-7, IL-8, IL-9, IP-10, MCP-1, MCP-3, MDC, MIP-1α, MIP-1β, PDGF-AA, PDGFAB-BB, RANTES, sCD40L, TGFα, TNFα, TNFβ, VEGF; - ICC was adjusted for age, gender, race/ethnicity, time intervals across blood collection | Multiplex | 3 | 2.86 to 15.26 years (median=4.49 years) |
| Yeh et al., 2019 [1] | Taipei | 20 | Range=20 to 40 | Subjects hospitalized in a chronic ward with stable psychiatric condition for more than six months | Plasma | -84 °C | - N=17; - IL-1β, IL-2, IL-4, IL-5, IL-6, IL-7, IL-8, IL-10, IL-12, IL-13, IL-17, G-CSF, GM-CSF, IFN-γ, MCP-1, MIP-1β, and TNF-α; - Crude ICC | BMIT | 2 | 14 days |
| Koelman et al., 2019 [2] | Germany;  European Prospective Investigation into Cancer and Nutrition (EPIC)-Potsdam study | 207 | Range=44.8 to 63.9;  Mean=56.7 | - Exclusion criteria included history of heart disease, impaired mobility, reported use of β-blockers, and had Systolic or diastolic blood pressure above 180 mm Hg or 110 mm Hg, respectively. - Male: n=83;   Female: n=124 | Plasma | -80 °C | - N=10; - IL-1β, IL-2, IL-4, IL-6, IL-8, IL-10, IL-12p70, IL-13, IFN-γ, and TNF-α; - Crude ICC | MSD | 2 | 4 months |
| Buxton et al., 2019 [3] | Mexico;  Pregnancy Research on Inflammation, Nutrition, & City Environment: Systematic Analyses (PRINCESA) | 104 | Range=20 to 35 | Pregnant women | Serum | Unknown | - N=20; - Eotaxin, IFNγ, IL-10, IL-12p40, IL-12p70, IL-17, IL-1rα, soluble IL-2 receptor alpha (sil-2rα), IL-1α, IL-1β, IL-2, IL-4, Il-6, IL-8, IP-10, MCP-1, MIP-1α, MIP-1β, TNFα, VEGF; - Crude ICC | Luminex | 3 times at gestational months 3, 5 and 7 | 4 months |
| Epstein et al., 2013 [4] | USA;  Multicenter AIDS Cohort Study (MACS) | 200 | Range=21.6 to 54.6 | Homosexual and bisexual men | Serum | -70 °C | - N=14; - ApoA1, sCD14, sgp130, sIL-6R, sIL-2Rα, sTNFR2, BAFF/BLyS, CXCL13, IFN-γ, IL-1β, IL-6, IL-8, IL-10, and TNF-α; - ICC was adjusted for age and race/ethnicity. | Luminex | 3 | Over 2 years |
| Biancotto et al., 2013 [5] | USA | 144 | Range=21 to 62;  Median=27 | - Healthy donors - Women: proportion=65%; Men: proportion=35% | Serum | -80 °C | - N=27; - IL-1β, IL-2, IL-4, IL-5, IL-6, IL-7, IL-8 (CXCL-8), IL-9, IL-10, IL-12p70, IL-13, IL-15, IL-17, eotaxin (CCL-11), IL-1Rα, FGF-β, G-CSF, GM-CSF, IFN-γ, IFN inducible protein-10 (IP-10; CXCL-10), MCP-1 (CCL-2), macrophage inflammatory protein 1α (MIP-1α; CCL-3), MIP-1β (CCL-4), PDGF-BB, RANTES (CCL-5), TNF-α, and VEGF - Paired t-test (or paired Wilcoxon rank sum test), spearmen correlation | Luminex | 2 | 1 week |
| Clendenen et al., 2010 [6] | Sweden;  Northern Sweden Health and Disease Study (NSHDS) cohort | 18 | Range=42 to 62 | Healthy women | Plasma | -80 °C | - N=30; - 22 cytokines and their soluble receptors (IL-1α, IL-1β, IL-1RA, IL-2, sil-2R, IL-4, IL-5, IL-6, sil-6R, IL-7, IL-8, IL-10, IL-12p40, IL-12p70, IL-13, IL-15, IL-17, TNFα, sTNF-R1, sTNF-R2, IFNα, IFNγ); - 8 growth factors (GM-CSF, EGF, bFGF, G-CSF, HGF, VEGF, EGFR, ErbB2); - Crude ICC | Luminex | 2 | 1 to 3 years (average of 2 years) |
| Hofmann et al., 2011 [7] | USA;  Prostate, Lung, Colorectal, and Ovarian (PLCO) Cancer Screening Trial | 28 | Range=55–70;  Mean=61 | - Cancer-free individuals; - Male: n=18;   Female: n=10 | Serum | -70 °C | - N=13; - IL-1β, IL-2, IL-4, IL-5, IL-6, IL-7, IL-8, IL-10, IL-12p70, IL-13, IFNγ, GM-CSF, and TNF-α; - Crude ICC | High-sensitivity Luminex | 2 to 3 times (study baseline, +1 year, and + 5 years) | Over 5 years |
| McKay et al., 2017 [8] | USA;  Multicenter AIDS Cohort Study (MACS) | 250 | Range=18.7 to 74.5;  Median=45.6 | Homosexual and bisexual men | Serum | Frozen | - N=22; - IL-1β, IL-2, IL-6, IL-10, IL-12p70, IFN-ɣ, GMCSF, TNF-α, and CCL11, CXCL10, CCL2, CCL13, CCL4, and CCL17; - ICC was adjusted for age. | MSD / Luminex | 5 | Median=18.3 years |
| Belzeaux et al., 2017 [9] | France | 20 | Unknown | Health subjects | Serum and plasma | -80 °C | - N=31; - Eotaxin, bFGF, G-CSF, IL-1RA, IL-9, IP-10, MCP-1, MIP-1α, MIP-1β, PDGF-BB, RANTES, IL-1α, IL-12p40, IL-16, TNF-β, GM-CSF, IFN-γ, IL-1β, IL-2, IL-4, IL-5, IL-6, IL-7, IL-8, IL-10, IL-12p70, IL-13, IL-15, IL-17, TNF, VEGF; - Crude ICC | Luminex | 4 | 30 weeks |
| Todd et al., 2013 [10] | USA | 42 | Health subjects: Range=24 to 61 years;  Mean=36 years;  Patients with cardiovascular risk factors: Range=42 to 96;  Mean=64 | - Apparently healthy subjects (n=25, 40% male); - Individuals attending a cardiovascular disease clinic (n=17, 9 females and 8 males) | Plasma | Frozen | - N=3; - IL-6, TNF-α, IL-17A. - Monthly biological variation (BV) and crude ICC were used to assess the variability of cytokines. | High sensitivity immunoassay (Erenna, Singulex, Inc.) | Health subjects: 6 times;  Patients: 3 times | Health subjects: over 6 weeks;  Patients: over 9 months |
| Navarro et al., 2012 [11] | USA | 62 | Range=20 to 40 | Subjects free of factors known to influence inflammation | Serum | -80 °C | - N=5; - IL-6, TNF-α, IL-8, sTNFRⅠ, sTNFRⅡ.; - Crude ICC | ELISA / high-sensitivity human cytokine panel / human soluble cytokine receptor panel | 4 | 4 to 6 months |
| Gu et al., 2009 [12] | USA;  New York University Women’s Health Study (NYUWHS) | 65 | Range=35 to 64;  Mean=50.8 | Premenopausal women (n=35);  Postmenopausal women (n=30) | Serum | -80 °C | - N=23; - IL-1α, IL-1β, IL-1RA, IL-2, IL-2Rα, IL-4, IL-5, IL-6, IL-6R, IL-7, IL-8, IL-10, IL-12p40, hsIL-12p70, IL-13, IL-15, IL-17,TNF-α, TNF-R1, TNF-R2, IFN-α, IFN-c, sCD40L; - ICC was adjusted for order of blood donation, blood storage time, age at blood donation, menopausal status, phase of menstrual cycle for premenopausal women, body mass index (BMI), race/ethnicity, medication use during a four-week period before the blood donation, alcohol consumption at baselin, and smoking status at baseline | Luminex | Premenopausal women: 3 times;  Postmenopausal women: 2 times | Over 2 years |
| Lee et al., 2007 [13] | China;  Shanghai Men’s Health Study (SMHS) | 48 | Range=40 to 74;  Mean=54.8 | Men lived in urban area of Shanghai and aged 40 to 74 years | Plasma | -70 °C | - N=12; - IL-1β, IL-6, IL-8, TNF-α, PAI-1, hsCRP, MCP-1, NGF, Leptin, Adiponectin, HGF, Resistin; - Crude ICC | Luminex | 4 | More than 1 year |
| Abbreviations: MSD, Meso Scale Discovery; BMIT, bead-based multiplex immunoassay technology. | | | | | | | | | | |

**References**

1. Yeh TC, Chu HT, Tsai CK, Chang HA, Yang FC, Huang SY, et al. Distinct Inflammation Biomarkers in Healthy Individuals and Patients with Schizophrenia: A Reliability Testing of Multiplex Cytokine Immunoassay by Bland-Altman Analysis. Psychiatry Investig. 2019;16(8):607-14.

2. Koelman L, Pivovarova-Ramich O, Pfeiffer AFH, Grune T, Aleksandrova K. Cytokines for evaluation of chronic inflammatory status in ageing research: reliability and phenotypic characterisation. Immun Ageing. 2019;16:11.

3. Buxton MA, Meraz-Cruz N, Sanchez BN, Gronlund CJ, Foxman B, Vadillo-Ortega F, et al. Air pollution and inflammation: Findings from concurrent repeated measures of systemic and reproductive tract cytokines during term pregnancy in Mexico City. Sci Total Environ. 2019;681:235-41.

4. Epstein MM, Breen EC, Magpantay L, Detels R, Lepone L, Penugonda S, et al. Temporal stability of serum concentrations of cytokines and soluble receptors measured across two years in low-risk HIV-seronegative men. Cancer Epidemiol Biomarkers Prev. 2013;22(11):2009-15.

5. Biancotto A, Wank A, Perl S, Cook W, Olnes MJ, Dagur PK, et al. Baseline levels and temporal stability of 27 multiplexed serum cytokine concentrations in healthy subjects. PLoS One. 2013;8(12):e76091.

6. Clendenen TV, Arslan AA, Lokshin AE, Idahl A, Hallmans G, Koenig KL, et al. Temporal reliability of cytokines and growth factors in EDTA plasma. BMC Res Notes. 2010;3:302.

7. Hofmann JN, Yu K, Bagni RK, Lan Q, Rothman N, Purdue MP. Intra-individual variability over time in serum cytokine levels among participants in the prostate, lung, colorectal, and ovarian cancer screening Trial. Cytokine. 2011;56(2):145-8.

8. McKay HS, Margolick JB, Martinez-Maza O, Lopez J, Phair J, Rappocciolo G, et al. Multiplex assay reliability and long-term intra-individual variation of serologic inflammatory biomarkers. Cytokine. 2017;90:185-92.

9. Belzeaux R, Lefebvre MN, Lazzari A, Le Carpentier T, Consoloni JL, Zendjidjian X, et al. How to: Measuring blood cytokines in biological psychiatry using commercially available multiplex immunoassays. Psychoneuroendocrinology. 2017;75:72-82.

10. Todd J, Simpson P, Estis J, Torres V, Wub AH. Reference range and short- and long-term biological variation of interleukin (IL)-6, IL-17A and tissue necrosis factor-alpha using high sensitivity assays. Cytokine. 2013;64(3):660-5.

11. Navarro SL, Brasky TM, Schwarz Y, Song X, Wang CY, Kristal AR, et al. Reliability of serum biomarkers of inflammation from repeated measures in healthy individuals. Cancer Epidemiol Biomarkers Prev. 2012;21(7):1167-70.

12. Gu Y, Zeleniuch-Jacquotte A, Linkov F, Koenig KL, Liu M, Velikokhatnaya L, et al. Reproducibility of serum cytokines and growth factors. Cytokine. 2009;45(1):44-9.

13. Lee SA, Kallianpur A, Xiang YB, Wen W, Cai Q, Liu D, et al. Intra-individual variation of plasma adipokine levels and utility of single measurement of these biomarkers in population-based studies. Cancer Epidemiol Biomarkers Prev. 2007;16(11):2464-70.


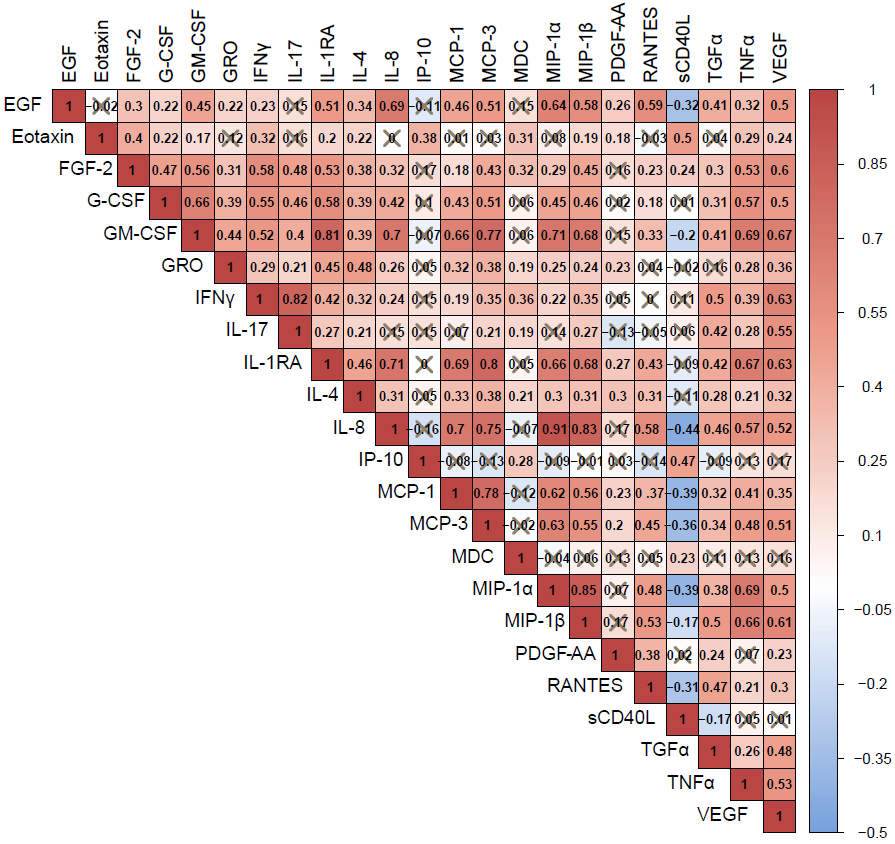
**Figure. S1.** Repeated measures correlation among different cytokines. The correlation coefficients are presented in the boxes in different colors. The dark grey crosses mean the correlations are not statistically significant (*p* > 0.05).


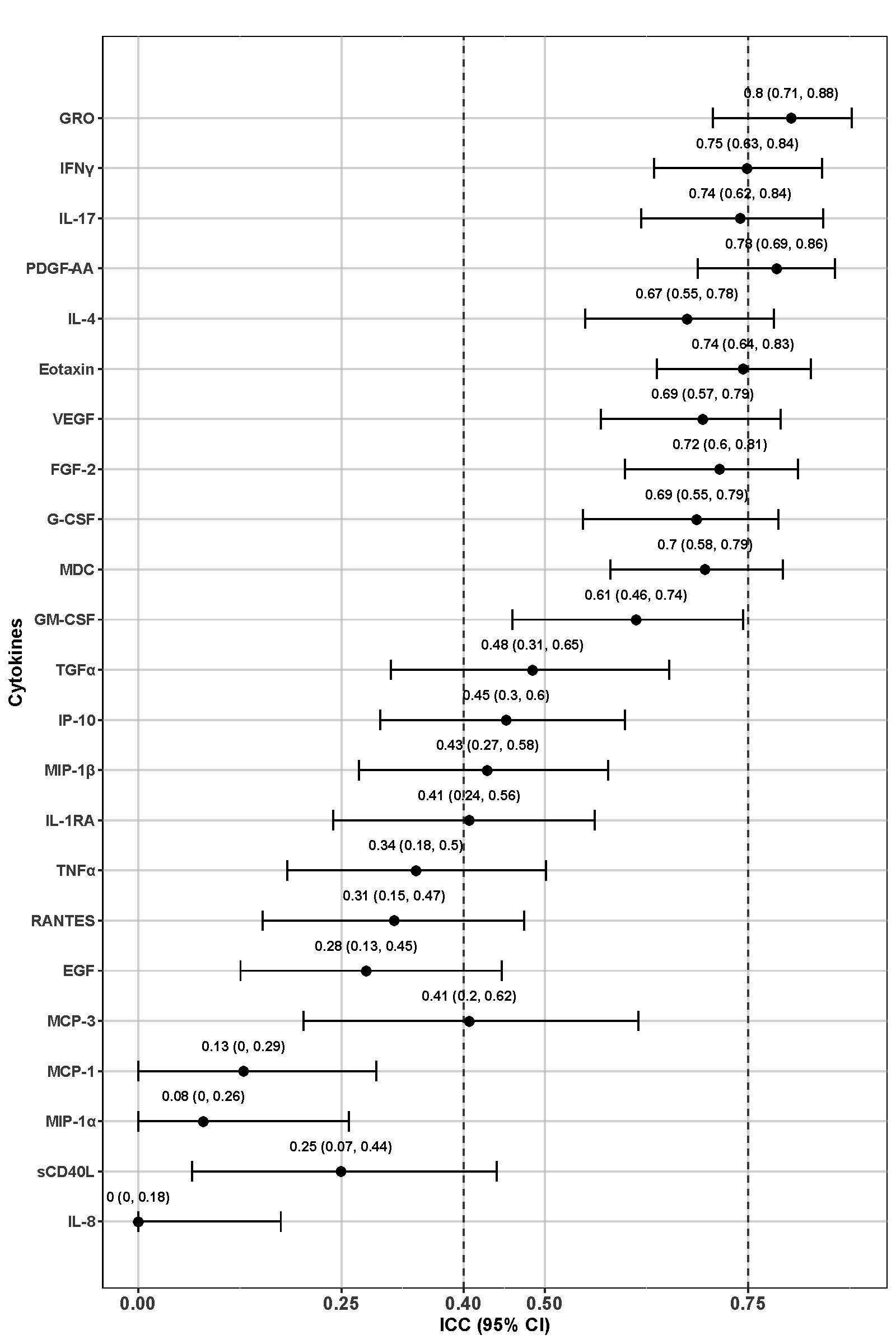


**Figure. S2.** ICC (95% CI) of cytokines by excluding the subjects with undetectable values. In the sensitivity analyses, ICC was separately estimated for specific cytokine when excluding subjects whose serum cytokines were undetectable. Samples with values between the LLOD and ULOD were regarded as detectable. The detectable rates were 100% in four cytokines of Eotaxin, MDC, IP10 and MCP1. The dots and horizontal bars represent the ICC and corresponding confidence intervals, respectively.


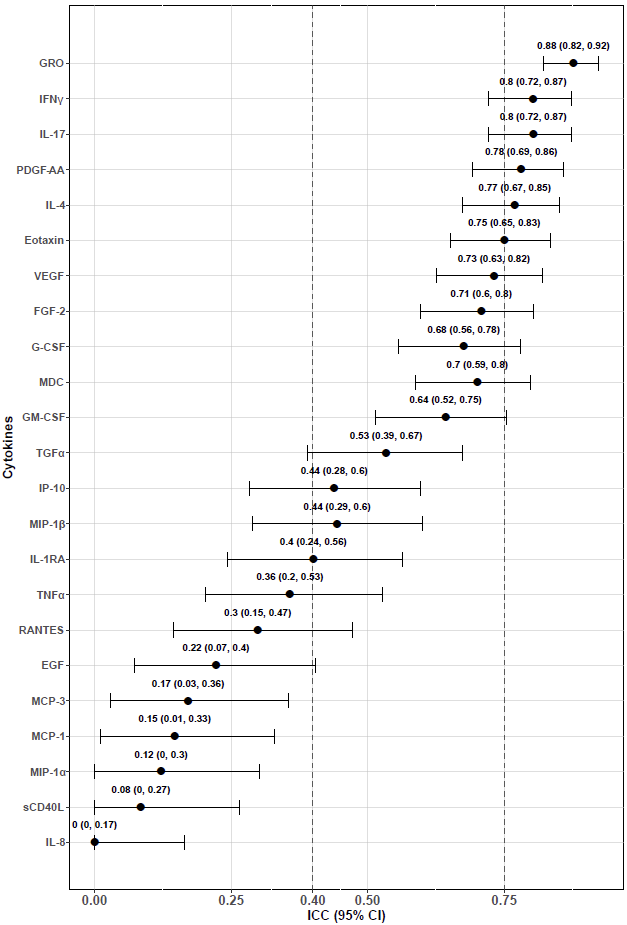


**Figure. S3.** ICC (95% CI) of cytokines when additionally adjusting for baseline comorbidities. In the sensitivity analyses, ICC was separately estimated for specific cytokine when additionally adjusting for the history of hypertension, diabetes and cardiovascular diseases. The dots and horizontal bars represent the ICC and corresponding confidence intervals, respectively.


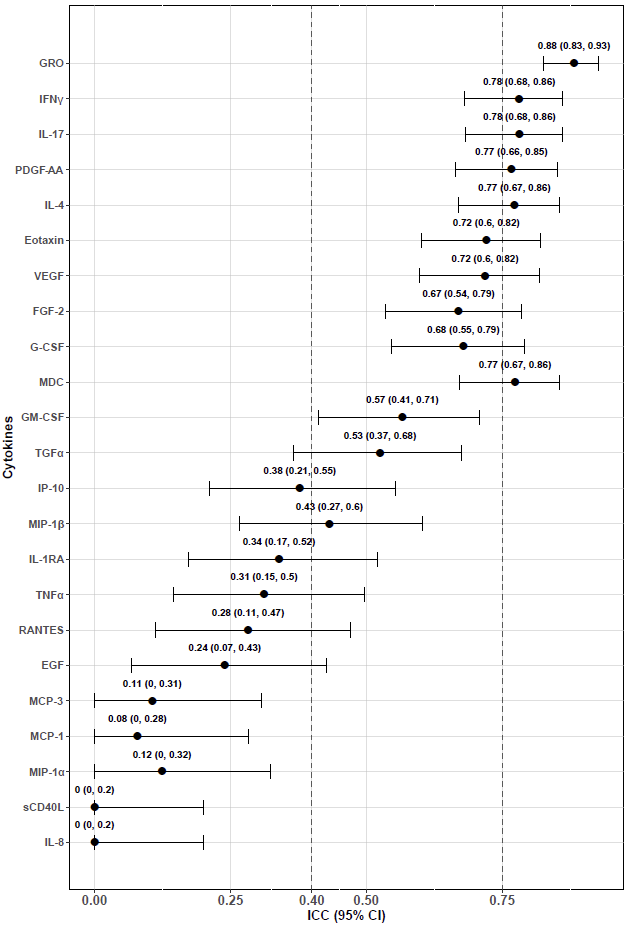


**Figure. S4.** ICC (95% CI) of cytokines when additionally adjusting for BMI categories. In the sensitivity analyses, ICC was separately estimated for specific cytokine when additionally adjusting for the BMI categories (normal weight, overweight, obese). The dots and horizontal bars represent the ICC and corresponding confidence intervals, respectively.


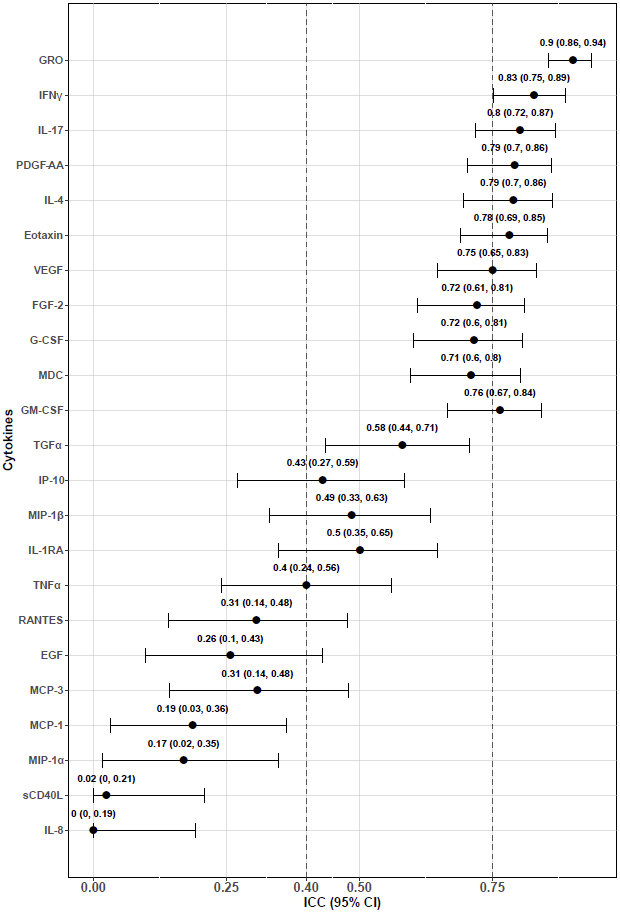


**Figure. S5.** ICC (95% CI) of cytokines when additionally adjusting for time of day of blood sample collection. In the sensitivity analyses, ICC was separately estimated for specific cytokine when additionally adjusting for time of day of blood sample collection. The dots and horizontal bars represent the ICC and corresponding confidence intervals, respectively.


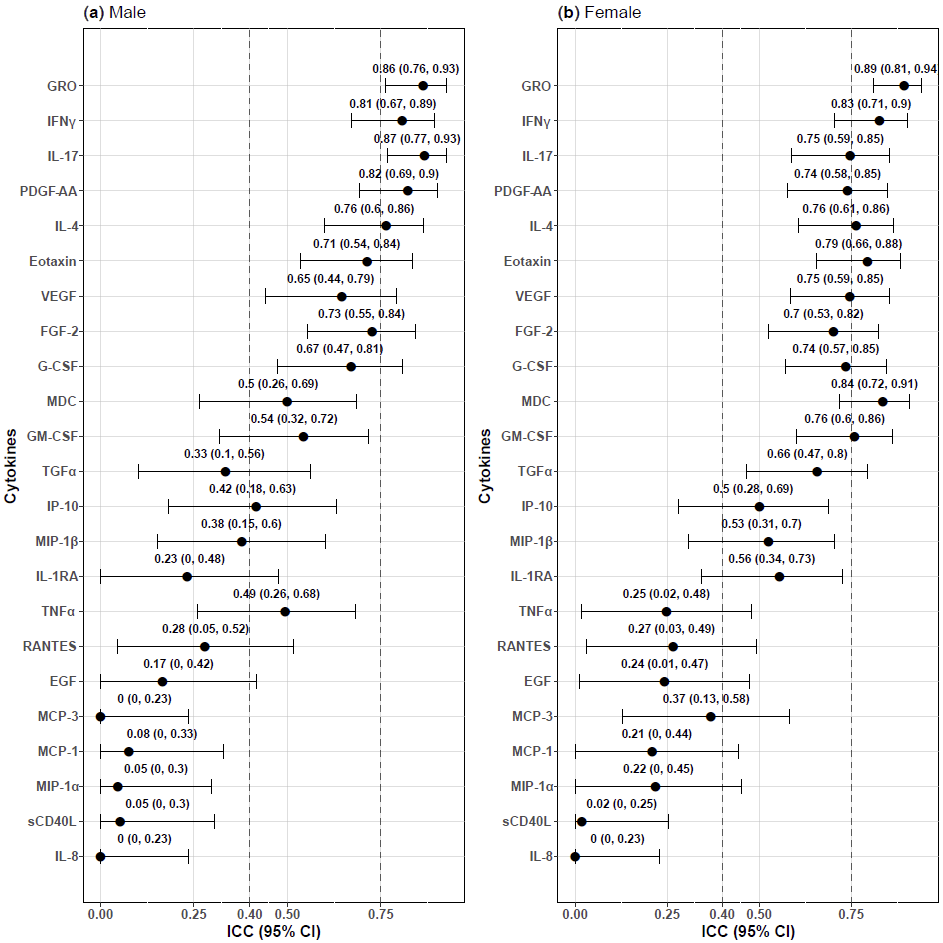


**Figure. S6.** ICC (95% CI) of cytokines stratified by sex. In the sensitivity analyses, ICC was separately estimated for specific cytokine by the stratification of sex, adjusting for the covariates of age at recruitment (years), race/ethnicity (non-Hispanic White, non-Hispanic Black, Hispanic) and time intervals across blood collection (years). The dots and horizontal bars represent the ICC and corresponding confidence intervals, respectively.
